# Endovascular Embolisation of Pulmonary Arteriovenous Malformation Using Amplatzer Vascular Plugs

**DOI:** 10.7759/cureus.24214

**Published:** 2022-04-17

**Authors:** Azeemuddin Muhammad, Zainab Rauf, Jehanzeb Shahid, Junaid Iqbal, Tanveer U Haq, Uffan Zafar

**Affiliations:** 1 Department of Radiology, Aga Khan University Hospital, Karachi, PAK; 2 Department of Radiology, Dow University of Health Sciences, Karachi, PAK

**Keywords:** secondary polycythemia, embolisation, endovascular interventions, amplatzer plug device, pulmonary avm

## Abstract

Pulmonary arteriovenous malformation (PAVMs) are abnormal communications between pulmonary arteries and veins. The rarity of their occurrence, coupled with the risks they pose, including brain abscess, embolic stroke, and myocardial infarction, mandates that they should not be overlooked in the differential diagnosis of patients presenting with haemoptysis, dyspnea, clubbing, cyanosis, hypoxemia, or epistaxis.

We present the case of a 41-year-old local female who presented to our hospital as an outpatient with decreased oxygen saturation (SpO_2_) of 70%-80% for the past two years with a final diagnosis of PAVM. The initial baseline workup showed polycythemia with a hemoglobin level of 19 mg/dL and raised hematocrit. She had extensive workup in the past two years for her polycythemia including gene mutation testing and cardiac workup which all turned out normal. Her chest X-ray (CXR) showed right lung opacity which was initially considered to be infective but it did not respond to antibiotic treatment. Later on, a CT scan of the chest was performed and findings were typical of a large PAVM which had two feeding arteries. The patient was referred to a cardiothoracic surgeon who sent the patient to the interventional radiology section for endovascular management.

The embolization procedure was then performed and both feeders were successfully embolised. After the procedure, the patient's SpO_2_ levels were restored to 95%-96%, and no post-procedure complications were noted.

## Introduction

Pulmonary arteriovenous malformations (PAVMs) are rare and debilitating vascular defects that provide a continuous high flow right-to-left shunt between pulmonary arteries and veins [1]. This condition affects around one in 50,000 individuals and its common symptoms include dyspnea, cyanosis, and clubbing [2]. The defect allows a proportion of the right ventricular stroke volume to bypass gas exchange. Despite impaired gas exchange, PAVM lesions of size less than 2 mm progress slowly and are usually asymptomatic due to the body’s compensatory mechanisms including polycythemia and increased cardiac output [1].

Most cases of PAVM are congenital, however, they usually manifest clinically during late adulthood [3]. A strong correlation exists between PAVM and hereditary hemorrhagic telangiectasia (HHT) which is an autosomal dominant condition. Approximately 70% - 75% of patients with PAVM are diagnosed with HHT [3]. Although rare, secondary or acquired PAVM arise from long-standing hepatic cirrhosis, metastatic thyroid carcinoma, mitral stenosis, tuberculosis, trauma, and infections such as actinomycosis and schistosomiasis [2].

PAVMs are usually overlooked in differential diagnoses due to their low occurrence despite critical complications such as brain abscesses and cerebrovascular accidents [1]. Subsequently, undiagnosed and untreated PAVMS are associated with up to 55% risk of mortality and morbidity compared to a dwindling 3% in treated patients [4]. Therefore, prompt treatment of PAVMs is essential.

Currently, trans-catheter embolization is the first-line treatment strategy for PAVMs [5]. Pregnancy, pulmonary hypertension, and renal impairment are relative contraindications to embolization [4]. Previously, surgical resection was the only available treatment, and even today, contraindicated embolization (which can be due to high risk of distal embolization) and large PAVMs with multiple complex feeding arteries require surgery [5]. We present the case of a patient with a large PAVM which was successfully treated by trans-catheter embolization without surgery.

## Case presentation

A 41-year-old local female presented to the cardiothoracic department of our tertiary care hospital with reduced oxygen saturation (SpO2) of 70%-80% for the past two years. Previously, the pulmonologist advised her to use supplemental oxygen. A baseline workup revealed polycythemia with a hemoglobin level of 19 mg/dl and raised hematocrit. It was being treated with venesection every six months. Further investigations were done namely gene mutation tests, cardiac screening, and chest X-ray (CXR). The CXR showed an opacity which was initially considered to be infectious. However, it did not respond to empiric antibiotic treatment. The patient had no typical signs of hypoxia secondary to PAVM like cyanosis or clubbing. Finally, a high-resolution computed tomography (HRCT) of the chest was performed which showed a lobulated density in the right mid lung zone, extending towards the right hilum, typical for a PAVM.

**Figure 1:**
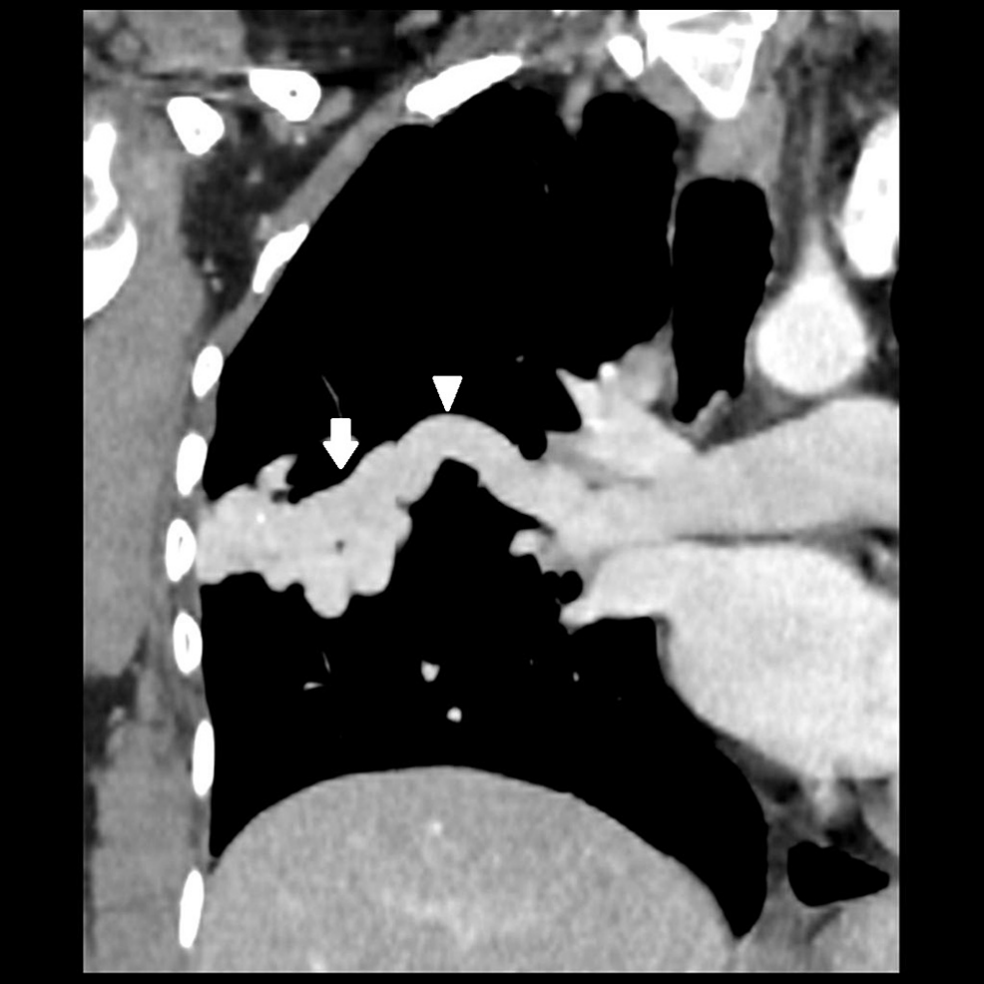
A homogeneously enhancing well-circumscribed lobulated vascular lesion in right mid lung zone (white arrow) which is extending up to the periphery of right lung with two arterial feeders identified arising from the upper lobe branch of right pulmonary artery and single draining vein to right upper lobe pulmonary vein (white arrowhead).

Upon presenting to us, a CT angiogram was performed which revealed a well-circumscribed lobulated density in the right upper lobe measuring 50 x 20 mm (Figure 1). Two arterial feeders measuring 6 mm and 5 mm, respectively were identified arising from the upper lobe branch of the right pulmonary artery with a single draining vein to the right upper lobe pulmonary vein. This led to the enlargement of the right-sided pulmonary vein and mild right heart enlargement secondary to high flow shunt.

**Figure 2:**
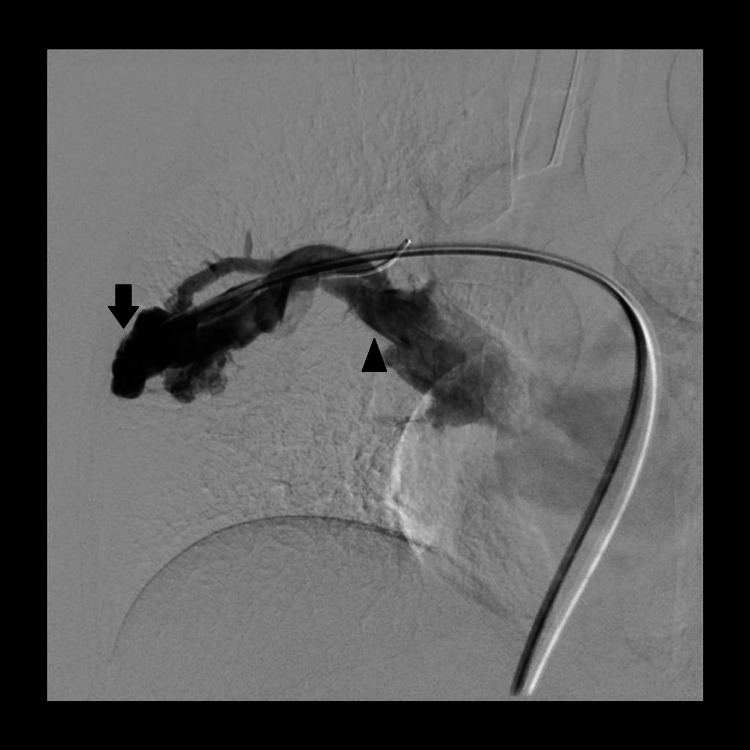
Digital subtraction angiogram of right upper lobe pulmonary artery shows an area of markedly dilated and tortuous vessels with nidi in the right middle lung zones (black arrow) which are supplied by feeders from right upper artery and draining via right superior pulmonary vein which is dilated (black arrowhead).

For the treatment of PAVM, the patient was referred to vascular interventional radiology for embolization and an endovascular embolization was scheduled. The procedure was carried out under general anaesthesia. The right common femoral vein was punctured at the groin and 5-Fr followed by an 8-Fr vascular sheath was placed using the Seldinger technique. The right pulmonary artery was initially cannulated with 4-Fr RDC (Boston Scientific, Natick, MA, USA) and then by a 4-Fr H1 catheter (Cordis Corp., Miami Lakes, FL, USA). At this stage, an angiogram was performed which demonstrated a large PAVM similar to that seen on CT (Figure 2). 

**Figure 3:**
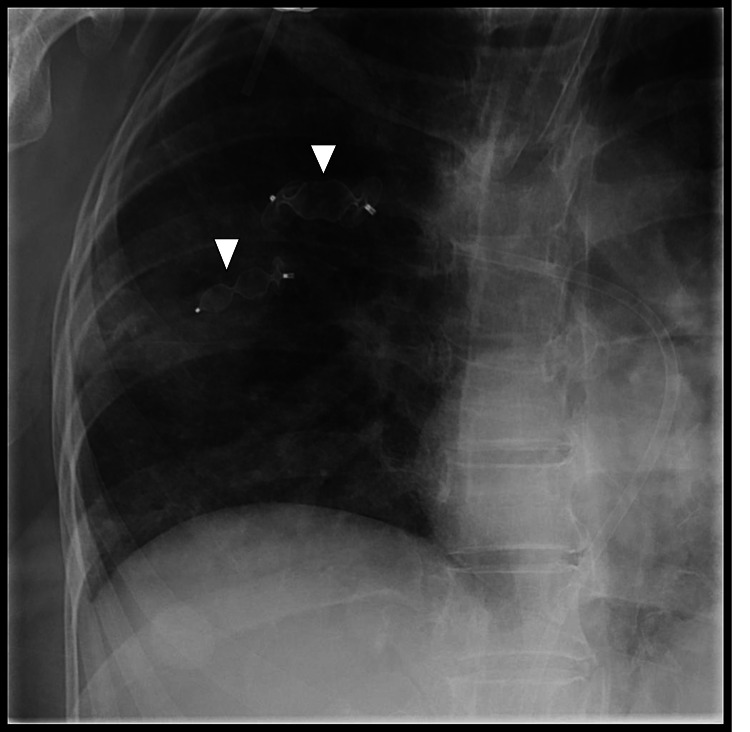
Plain radiograph image showing deployment of two Amplatzer vascular plugs (white arrowheads), one in each arterial feeders.

**Figure 4:**
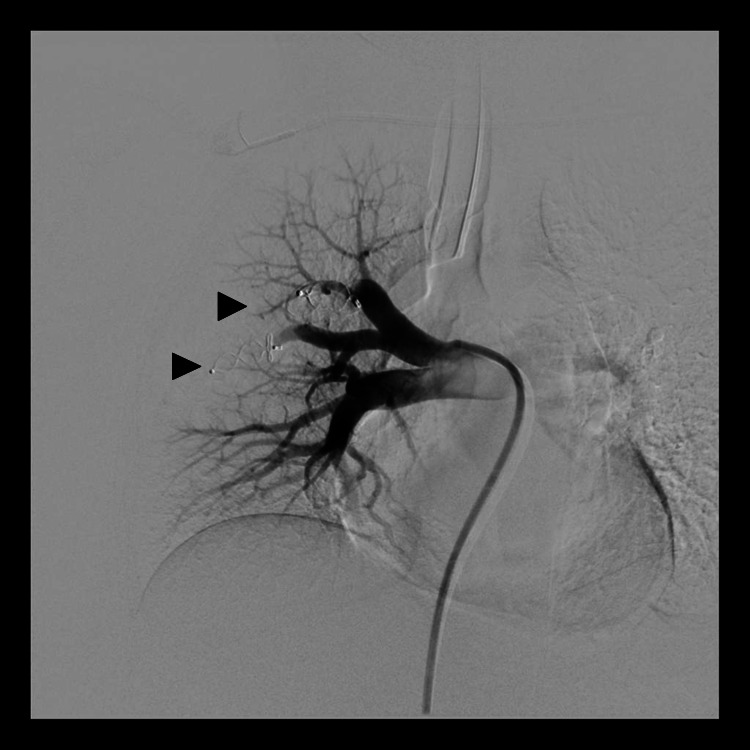
Digital subtraction angiogram, post embolization run shows complete occlusion of both arterial feeders (black arrowheads) representing successful embolization.

Selective cannulation of the arterial feeders was carried out; a long 6-Fr guiding sheath was introduced over the exchange length Amplatz wire and subsequently, both the feeders were embolized using two 7 mm x 8 mm and 12 mm x 8 mm Amplatzer vascular plugs (AGA Medical Corporation, Golden Valley, MN, USA), respectively (Figure 3). Post-embolization runs showed complete occlusion of the arterial feeders with no residual visible AVM (Figure 4). 

After the procedure, the patient's SpO_2_ levels were restored to 95%-96% and the post-procedure recovery remained unremarkable. The patient was discharged the next day in stable condition.

**Figure 5:**
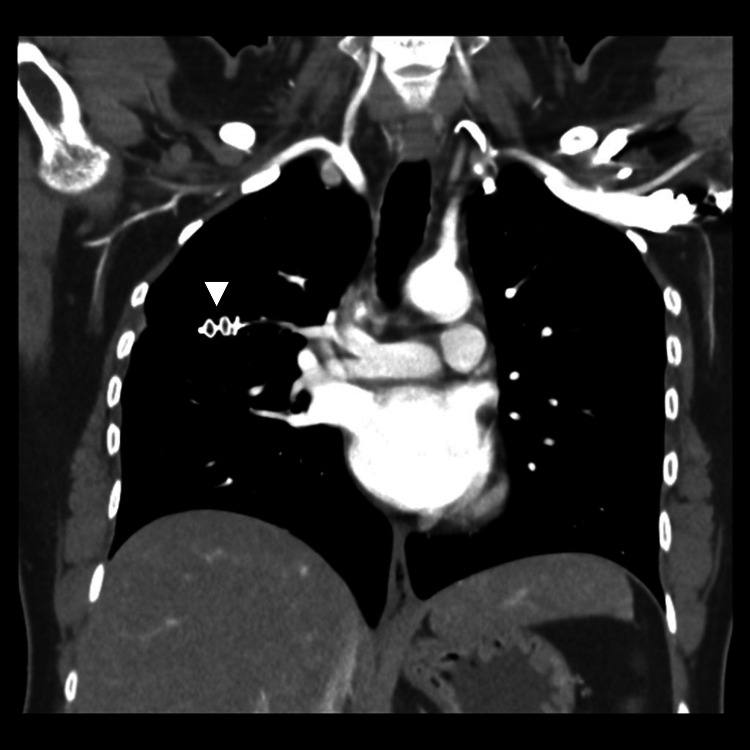
Coronal CT angiogram images done on two-month follow-up showing a vascular Amplatzer plug in place (white arrowhead) without visualization of any residual filling of arteriovenous malformation.

Follow up CT after two, six, and fifteen months months showed no residual AVM (Figure 5).

## Discussion

First described in 1987 by T. Churton, PAVM is a condition consisting of an abnormal connection between the pulmonary artery and pulmonary vein [1] and has a strong association with HHT [3]. Ideally, PAVMs are supposed to be a part of differential diagnosis in patients presenting with haemoptysis, dyspnea, clubbing, cyanosis, hypoxemia, and/or epistaxis [2]. According to two reports by the Mayo Clinic, only 68 [6] and 38 [7] patients were reported to have this condition over the course of more than 25 years. Consequently, the rarity of this disease coupled with the incomplete knowledge of its pathophysiology has resulted in commonly missed diagnosis of PAVMs.

Our case highlights a similar case of missed diagnosis for a minimum of two years. Our patient was asymptomatic and had venesection on multiple occasions to counter her elevated hemoglobin levels which proved unfruitful. She was prescribed supplemental oxygen to correct her consistently low oxygen saturation. However, using supplemental oxygen is a tedious process that does not fix the underlying condition. Eventually, the diagnosis of PAVM was made by a CT chest which is a good screening tool for PAVMs.

Multiple treatment strategies are available for the treatment of PAVMs. Historically, PAVMs were treated with invasive surgery by open excision, ligation, lobectomy, or segmentectomy [8]. However, this surgery carried a significant risk of morbidity and prolonged hospitalization. Invasive surgery also carried the risk of additional complications related to general anesthesia, loss of lung parenchyma, surgical site infection, and hospital-acquired pneumonia in the postoperative period [8]. Currently, endovascular embolization remains the treatment of choice for PAVMs as the technique is minimally invasive, requires very little post-procedure care, and can be easily repeated if needed. All PAVMs greater than 3 mm in size need treatment regardless of the patient's clinical symptoms [9].

Surgical management is now reserved only for those cases which are not suitable for endovascular management including large or centrally placed AVMs. A large study conducted in Japan by Nagano et al. (2017) concluded that trans-catheter embolization posed a lower risk of complications and shorter post-operative hospital stay as compared to the surgery [10].

In the early days, endovascular embolization was achieved by using detachable balloons which were later removed from the market due to the risk of deflation [11]. Liquid embolic agents like histoacryl glue and Onyx are also not preferred as most treatable AVMs have large feeding arteries with high flow and these embolization agents may not hold steady [12]. Currently, the preferred treatments are vascular coils and vascular plugs. Advantages of coils include a wide range of available sizes, better outcomes in small arterial feeders, and better maneuverability due to greater flexibility of the device. On the other hand, the disadvantage of coils is the risk of distal migration, especially in the case of pushable coils or large feeding arteries. Advantages of vascular plugs include better outcomes in large feeding arteries due to a low risk of distal migration [13]. Treatment choice between vascular plugs and coils is multifactorial including the size of the feeding artery and anatomy of AVM. Therefore, the final choice of the device to be used still varies from case to case. A combined approach with vascular plugs and coils can also be used in large and complex AVMs that have a high risk of recanalization [14].

The most commonly encountered complication with endovascular embolization is pleuritic chest pain which is often short-lived and can be managed conservatively [15]. Infrequent, but one of the most feared complications of endovascular treatment is distal coil migration. Most of the distally dislodged coils can be snared out without posing any significant risk to patient outcome [16]. Air embolization is another possible complication and air is prone to especially enter the left coronary artery which is anteriorly situated. It causes chest pain, temporary ECG changes, and bradycardia which usually responds well to sublingual nitroglycerine [17]. Additionally, continuous cardiac monitoring and a ready crash cart with atropine should be available to treat bradycardia and hypotension.

Unlike most rare conditions, treatment for PAVM has a high success rate [18] and rarely results in complications, ensuring a good quality of life and a marked reduction of morbidities including brain abscesses in treated patients [4]. Considering PAVM as a differential diagnosis in patients with unexplained symptoms of polycythemia and low oxygen saturation, with or without a history of HHT, can reduce the chances of unwanted emergencies and should be exercised as a routine.

## Conclusions

Owing to their rarity, PAVMs are sparsely seen in current literature, especially in developing countries like Pakistan. This can be attributed to multiple reasons including lack of resources, education, lack of access to tertiary healthcare setups, and even general awareness about elevated hemoglobin levels and its implications. Continued research on PAVMs along with improvement in current screening methods will rectify this situation and better accommodate idiopathic cases. 
